# Identification of Key Ubiquitination Sites Involved in the Proteasomal Degradation of AtACS7 in *Arabidopsis*

**DOI:** 10.3390/ijms25052931

**Published:** 2024-03-02

**Authors:** Xianglin Tang, Ran Liu, Yuanyuan Mei, Dan Wang, Kaixuan He, Ning Ning Wang

**Affiliations:** Tianjin Key Laboratory of Protein Sciences, Department of Plant Biology and Ecology, College of Life Sciences, Nankai University, Tianjin 300071, China

**Keywords:** AtACS7, ubiquitination, proteasomal degradation, LC-MS/MS

## Abstract

The gaseous hormone ethylene plays pivotal roles in plant growth and development. The rate-limiting enzyme of ethylene biosynthesis in seed plants is 1-aminocyclopropane-1-carboxylic acid (ACC) synthase (ACS). ACS proteins are encoded by a multigene family and the expression of *ACS* genes is highly regulated, especially at a post-translational level. AtACS7, the only type III ACS in *Arabidopsis*, is degraded in a 26S proteasome-dependent pathway. Here, by using liquid chromatography–mass spectrometry/mass spectrometry (LC-MS/MS) analysis, two lysine residues of AtACS7, lys285 (K285) and lys366 (K366), were revealed to be ubiquitin-modified in young, light-grown *Arabidopsis* seedlings but not in etiolated seedlings. Deubiquitylation-mimicking mutations of these residues significantly increased the stability of the AtACS7^K285RK366R^ mutant protein in cell-free degradation assays. All results suggest that K285 and K366 are the major ubiquitination sites on AtACS7, providing deeper insights into the post-translational regulation of AtACS7 in *Arabidopsis*.

## 1. Introduction

As a significant gaseous hormone, ethylene has crucial effects on many developmental and physiological processes of plants, such as seed germination [1], root hair formation [2], floral transition [3], fruit ripening [4], senescence [5], abscission [6], and responses to biotic and abiotic stresses [7,8,9,10,11,12]. Critical to the functions of ethylene is the stringent regulation of its biosynthesis. Excessive ethylene not only causes drastic morphological changes but also leads to premature leaf senescence or even plant death [13,14]. However, if ethylene biosynthesis is severely hindered, plants often display stunted leaf development and even embryonic lethality [15]. Therefore, the biosynthesis of ethylene in plants is precisely and tightly controlled to ensure a normal growth and development process throughout their life.

The biosynthetic pathway of ethylene has been extensively studied [16,17]. The first step in ethylene biosynthesis is the conversion of *S*-adenosylmethionine (SAM) to 1-aminocyclopropane-1-carboxylic acid (ACC) by the rate-limiting enzyme ACC synthase (ACS). The expressional levels and catalytic activities of different ACS members determine the production level of endogenous ethylene [15]. In *Arabidopsis*, there are eight homologous genes encoding active ACS proteins, which are classified into three types based on the presence of particular sequences at the C terminus [15,18]. Compared to type I and type II ACSs, type III ACS proteins have the shortest C-terminal extensions and lack the specific phosphorylation sites and target of ETO1 (TOE) domain, which are crucial for the post-translational regulations of type I and type II ACSs [19]. This implies that an intriguing mechanism underlies the regulation of type III ACS protein stability and abundance.

AtACS7 is the only type III ACS isozyme in *Arabidopsis* and functions in multiple developmental processes. It has been reported that AtACS7 is degraded in a 26S proteasome-dependent manner [20,21]. The covalent attachment of a polyubiquitin chain to a lysine residue on a target protein is a prerequisite for 26S proteasome-dependent degradation [22]. However, the key ubiquitination modified site(s) involved in the proteasomal degradation of AtACS7 have not been identified. In this study, using liquid chromatography-mass spectrometry/mass spectrometry (LC-MS/MS) analysis, two lysine residues, lys285 (K285) and lys366 (K366), were revealed to be the major ubiquitination sites of AtACS7 from the light-grown *Arabidopsis* seedlings. Using a cell-free assay, we demonstrated that, when both K285 and K366 were mutated to arginine, the proteasomal degradation of AtACS7 was significantly retarded. These results confirm that K285 and K366 are major ubiquitination sites of AtACS7 and play important roles in the regulation of AtACS7 protein stability.

## 2. Results

**Figure 1:**
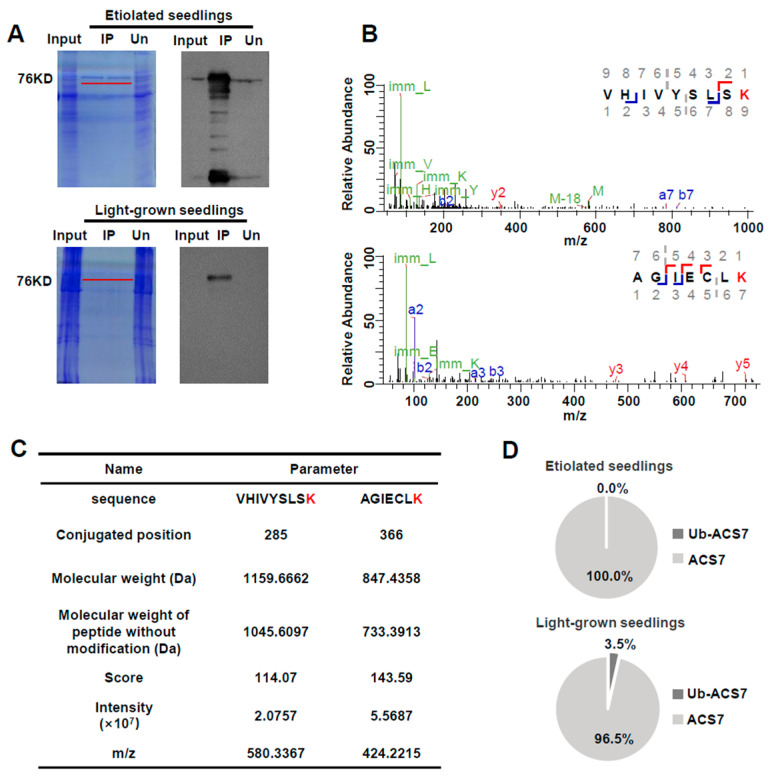
Identification of ubiquitination sites of AtACS7 by liquid chromatography-mass spectrometry/mass spectrometry (LC-MS/MS). (**A**) SDS-PAGE and Western blot analysis were used for the AtACS7 protein enriched for LC-MS/MS from either etiolated or light-grown *35S:AtACS7-eGFP* transgenic *Arabidopsis* seedlings. Proteins subjected to MS analysis after GFP trap enrichment are indicated by red lines. Un: unbounded proteins after immunoprecipitation. (**B**,**C**) LC-MS/MS spectrum of peptide segments ^277^VHIVYSLSK^285^ and ^360^AGIECLK^366^. Additionally, the K285 and K366 proteins were identified as the ubiquitin sites. Two ubiquitinated lysines are shown in red. (**D**) The proportions of ubiquitinated AtACS7 peptides in the AtACS7 protein enriched from the etiolated and light-grown *35S:AtACS7-eGFP* seedlings are shown.

AtACS7 is more stable in etiolated seedlings but is prone to be degraded via ubiquitin–26S proteasome pathway in light-grown seedlings [20,23]. To acquire enough AtACS7 protein for the identification of ubiquitination sites on AtACS7 protein by LC-MS/MS, line 3 of the *35S:AtACS7-eGFP* transgenic *Arabidopsis*, previously reported by our group [20], was selected. Total proteins were extracted from 4-day-old etiolated seedlings and 3-week-old light-grown seedlings of *35S:AtACS7-eGFP* transgenic Arabidopsis, respectively, and the AtACS7 protein was enriched using GFP-Trap coupled to magnetic beads (Figure 1A). Consistent with the previous study [20], the accumulation of the AtACS7 protein in etiolated seedlings was significantly higher than in light-grown seedlings (Figure 1A).

The enriched proteins were digested with trypsin and analyzed using LC-MS/MS. The mass error for precursor ions was set to 20 ppm. A total of 153 and 63 AtACS7 peptides were obtained from protein samples enriched from the etiolated and light-grown seedlings of the AtACS7-overexpressing line, among which 36 and 32 peptides, containing the lysine residue with peptide score more than 100, were identified, respectively. Two target peptides, ^277^VHIVYSLSK^285^ and ^360^AGIECLK^366^, were obtained only from the samples prepared from the light-grown seedlings instead of the etiolated seedlings. Each of the fragments harbored a ubiquitin-modified lysine residue, namely K285 and K366, respectively (Figure 1B–D). These results suggest that the AtACS7 protein in light-grown seedlings is more prone to being ubiquitin-modified at these two lysine residues.

**Figure 2:**
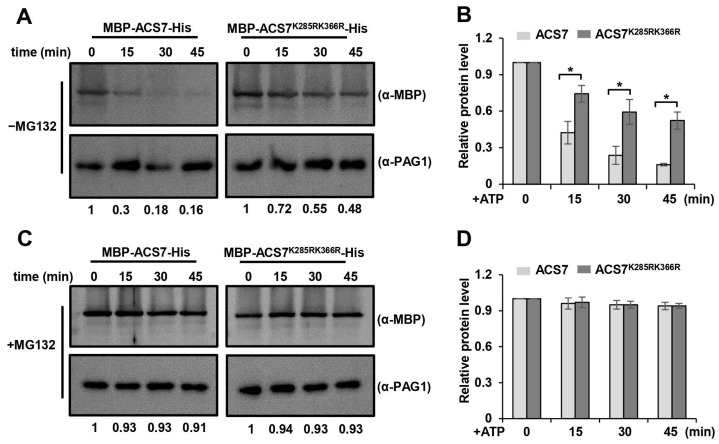
The degradation of AtACS7 and AtACS7^K285RK366R^. Degradations of AtACS7^K285RK366R^ and its wild-type control were examined in the cell-free degradation assays without (**A**,**B**) or with (**C**,**D**) MG132 treatment. The purified MBP-AtACS7-His or MBP-AtACS7^K285RK366R^-His mutant protein was incubated with total protein extracted from 10-day-old light-grown *Arabidopsis* seedlings. The MBP-AtACS7-His and MBP-AtACS7^K285RK366R^-His proteins at the indicated time points were determined by Western blot analysis using an anti-MBP antibody. Anti-PAG1 was used as a sample loading control. The intensity ratios between AtACS7 and the PAG1 band in each lane were normalized to time the point of 0 min (value set to 1) and are shown under the blots. Representative gel images of the three biological replicates are shown in (**A**,**C**), and the relative levels of MBP-AtACS7-His and MBP- AtACS7^K285RK366R^-His are shown in (**B**,**D**). Error bars represent SD from three independent assays. (Student’s *t* test, * *p* < 0.05). min, minutes.

To confirm the ubiquitination sites on AtACS7 and validate their effects on AtACS7 degradation, we replaced both lysine residues identified above with arginine (R) to mimic deubiquitylation on these positions. The wild-type and mutated forms of AtACS7 were fused with maltose binding protein (MBP) and histidine (His), generating MBP-AtACS7-His and MBP-AtACS7^K285RK366R^-His, respectively. After purification using affinity chromatography, the site-directed mutant proteins were incubated with total proteins extracted from the wild-type *Arabidopsis* seedlings, and protein stability was compared with wild-type AtACS7 in a cell-free assay. The results show that the wild-type MBP-AtACS7-His protein was degraded quickly and only less than 20% of the AtACS7 protein remained after a 45-min incubation. However, MBP-AtACS7^K285RK366R^-His was degraded much more slowly, and there was nearly 50% of the mutated AtACS7 protein left at the time point of 45 min, demonstrating that replacing the two lysine residues with arginine markedly retarded the degradation of AtACS7 (Figure 2A,B). The degradations of MBP-AtACS7-His and MBP-AtACS7^K285RK366R^-His were both blocked by the proteasome inhibitor MG132 (Figure 2C,D). Taken together, our data indicate that lys285 and lys366 are major ubiquitin-modified sites on the AtACS7 protein, and ubiquitin attachment to these sites significantly promotes AtACS7 protein degradation by the 26S proteasome.

## 3. Discussion

Ubiquitin proteasome system (UPS) is an efficient and effective way to control the abundance of cellular proteins in eukaryotes. The covalent attachment of one or more ubiquitin molecules to internal lysine residues is necessary for the degradation of target proteins. AtACS7 was previously reported to be degraded in the ubiquitin-26S proteasome-dependent pathway, implying that internal lysine residues of AtACS7 can be modified by ubiquitin molecules. However, previous studies found that K435, the only lysine residue in the C-terminal of AtACS7, did not regulate the stability of AtACS7 [21]. Here, we demonstrated that K285 and K366 are major ubiquitination sites of AtACS7 and play a significant role in the regulation of AtACS7 protein stability and abundance.

E3 ligase recognizes the target and transfers the active ubiquitin molecule to the internal lysine residues of the substrate protein. It was reported that RING-type E3 ligase XB3 ortholog 2 in *Arabidopsis thaliana* (XBAT32) was involved in the proteasomal degradation of AtACS7 [21]. Whether K285 or K366 is the target site of XBAT32 remains to be answered. The N-terminal 14 residues that do not contain lysine residue are also involved in the proteasomal degradation of AtACS7 itself [20]. Nevertheless, whether the N-terminus-mediated degradation of AtACS7 is related to the ubiquitin-modified lysine residues remains yet unknown. In addition, though the proteasomal degradation of AtACS7 was significantly retarded, it was not completely suppressed by mutations at both K285 and K366. This observation implies that there may be other ubiquitin-modified residue(s) to be identified or other degradation pathway(s) involved in the proteasomal degradation of AtACS7. Identification of K285 and K366 in the current study provided deeper insights into the complicated mechanisms underlying the post-translational regulation of AtACS7.

AtACS7 has dual enzymatic activities. It can catalyze either the formation of ethylene precursor ACC as an ACS or the cleavage of the C_β_-S bond in cystine as a carbon–sulfur lyase (C-S lyase, CSL) [24]. Both ACS and CSL enzymes require pyridoxal-5′-phosphate (PLP) as a cofactor for their catalysis [24]. The K285 residue of AtACS7 mediates the formation of a Schiff base linkage between the aldehyde group of PLP and the amine acid substrate, thereby generating an external aldimine. This residue can also extract the C_α_ proton of _L-_cystine and transfer it to the S_γ_ atom, thereby breaking the bond between C_β_ and S_γ_ of _L-_cystine, and thus producing a quinonoid intermediate in the C_β_-S lyase activity of AtACS7 [24]. The K285 residue, therefore, is not only essential for the enzymatic activities but also affects the stability of AtACS7. Whether the ubiquitination at K285 has any effect on the enzymatic activities of AtACS7 is worthy of further study.

## 4. Materials and Methods

### 4.1. Plant Materials and Growth Condition

*Arabidopsis* used in this study were in the Columbia-0 (Col) ecotype background. Line 3 of the *35S:AtACS7-eGFP* transgenic Arabidopsis used in this study was characterized previously by our group [20]. The *Arabidopsis* seeds were sterilized in 10% (*v*/*v*) sodium hypochlorite. The etiolated seedlings were grown on half-strength Murashige and Skoog (MS) medium containing 1% sucrose at 22 °C in the dark for 4 days. The light-grown seedlings were grown on half-strength MS medium containing 1% sucrose at 22 °C for 10 days and then transferred to soil for another 11 days under long-day conditions (16 h light, 8 h dark).

### 4.2. Construction and Purification of MBP-AtACS7-His and MBP-AtACS7^K285RK366R^-His

The cDNA fragment encoding AtACS7 protein was PCR-amplified with *ACS7-F* and *ACS7-R* primers and cloned into *pMAL-c5X* (NEB, Beijing, China) via restriction sites *Not* I and *EcoR* I (Takara, Dalian, China) to generate the MBP-AtACS7-His construct. The MBP-AtACS7^K285RK366R^-His construct was generated by overlapping PCR [25]. First, two fragments were generated using MBP-AtACS7-His as a template and with *ACS7-F* and *ACS7^K285R^-R*, *ACS7^K285R^-F* and *ACS7-R* as primers. The products were cloned into *pMAL-c5X* by In-Fusion Cloning Kit (Beyotime, Shanghai, China) to generate the *MBP-AtACS7^K285R^-His* construct. Then, the MBP-AtACS7^K285RK366R^-His construct was obtained by the same strategy using the *MBP-AtACS7^K285R^-His* as a template and *ACS7-F*, *ACS7^K366R^-R*, *ACS7^K366R^-F*, and *ACS7-R* as primers. All primers used in this study are listed in Supplemental Table S1.

Plasmids were transformed into *E. coli* BL21 [Rosetta ^TM^2 (DE3) plysS] (Biomed, London, UK). Recombinant proteins were purified via affinity chromatography using His-Trap FF columns (GE Healthcare, Buckinghamshire, UK) according to the manufacturer’s instructions.

### 4.3. Protein Extraction and IP Coupled with LC-MS/MS Analysis

Four-day-old etiolated seedlings and 3-week-old light-grown seedings of *35S:AtACS7-eGFP* were harvested and ground in liquid nitrogen to a fine powder. Total soluble proteins were extracted using extraction buffer (50 mM Tris–HCl pH 7.5, 150 mM NaCl, 0.5 mM EDTA, 1% Triton X-100 and 1 × complete protease inhibitor cocktail) and incubated with GFP-Trap magnetic beads (GFP-Trap MA) for 2.5 h at 4 °C. The beads were then washed five times with washing buffer (10 mM Tris-HCl pH 7.5, 150 mM NaCl, 0.5 mM EDTA, and 0.05% NP-40) and eluted with 80 μL protein elution buffer (120 mM Tris-HCl pH 6.8, 20% glycerol, 4% SDS, 0.04% bromophenol blue, 10% β-mercaptoethanol).

Ten microliters of the eluted sample was separated by 12% SDS-PAGE and transferred to a polyvinylidene fluoride (PVDF) membrane. The AtACS7-eGFP was detected using a GFP antibody (Abcam, Cambridge, UK). The remaining sample was subjected to SDS–PAGE, followed by Coomassie blue staining, and the single band of AtACS7 was cut out to identify ubiquitin-modified sites by LC-MS/MS (Beijing Bio-Tech Pack Technology Company Ltd., Beijing, China). The raw MS files were analyzed and searched against target protein database based on the species of the samples using Byonic. The parameters were set as follows: the protein modifications were carbamidomethylation (C) (variable), oxidation (M) (variable), GlyGly (K) (variable); the enzyme specificity was set to trypsin; the maximum missed cleavages were set to 2; the precursor ion mass tolerance was set to 20 ppm, and MS/MS tolerance was 0.02 Da. Only highly confident identified peptides were chosen for downstream protein identification analysis. Protein identifications were accepted if they could be established at >95% probability and contained at least two identified peptides.

### 4.4. Cell-free Protein Degradation Assay

Ten-day-old light-grown seedings were harvested and ground in liquid nitrogen to a fine powder. Three volumes of in vitro degradation buffer (25 mM Tris–HCl pH 7.5, 10 mM NaCl, 10 mM MgCl_2_, 1% Triton X-100, 5 mM dithiothreitol, and 10 mM ATP) were added and lysed on ice for 30 min. After centrifugation at 12,000 rpm for 15 min at 4 °C, the supernatants were incubated with 1.5 μg MBP-AtACS7-His or MBP-AtACS7^K285RK366R^-His at 28 °C for the indicated times. Reactions were blocked by adding a sample buffer (250 mM Tris-HCl pH 6.8, 50% glycerol, 10% SDS, 0.5% bromophenol blue, 5% β-mercaptoethanol). Then, the samples were separated by 12% SDS-PAGE and transferred to a PVDF membrane. The AtACS7 protein was detected using an anti-MBP antibody (NEB, Beijing, China) and PAG1 was detected using an anti-PAG1 antibody (Abcam, Cambridge, UK). Image J (http://rsb.info.nih.gov/ij/, last accessed 19 February 2024) software was used for quantification of the protein band intensities from immunoblots.

## Data Availability

Data are contained within the article and Supplementary Materials.

## Supplementary Materials

The following supporting information can be downloaded at: https://www.mdpi.com/article/10.3390/ijms25052931/s1.
