# Supplementary material for: Identification of Key Ubiquitination Sites Involved in the Proteasomal Degradation of AtACS7 in Arabidopsis
Source: Int J Mol Sci. 2024 Mar 2;25(5):2931. doi: 10.3390/ijms25052931 (PMC10931761; doi:10.3390/ijms25052931)
Supplement: Supplementary file 1 [file ijms-25-02931-s001.zip › ijms-2860818-supplementary.pdf]

Supplementary Table S1. Primers used to generate constructs. The underlined sequences in primers show introduced restriction enzyme sites. Mutation sites are highlighted in red.

Primers used to generate constructs		
Construct	Primers	Sequence (5' to 3')
MBP-ACS7-His	MBP-ACS7-His-F	CACATATGTCCATGGGCGGCCGCATGGGTCTTCCTCTAATGAT
	MBP-ACS7-His-R	CTGCAGGGAATTCGTGATGATGATGATGATGAAACCTCCTTCGTCG
MBP-ACS7^{K285RK366R}-His	ACS7^{K285R}-F	AGTCTCTCCAGGGATCTTGGTCTTCCTGGT
	ACS7 ^{K285R}-R	ACCAAGATCCCTGGAGAGACTGTAGACGAT
	ACS7^{K366R}-F	GAGTGTTTGAGAGGGAACGCAGGGCTATTT
	ACS7 ^{K366R}-R	TGCGTTCCCTCTCAAACACTCAATCCCTGC
